# The Nursing Supplement

**Published:** 1890-03-15

**Authors:** 


### "The Hospital" Nursing Mirror.

Being the Extra Nursing Supplement of "The Hospital" Newspaper.

All Contributions for this Supplement should be addressed to the Editor, The Hospital, 140, Strand, London, W.C., and should have the word "Nursing" plainly written in left-hand top corner of the envelope.

### En Passant.

JERSEY INSTITUTION.—The Rev. G. O. Balleine presided at the annual meeting of this institution. The report shows a falling off in annual subscriptions, and also in the nurses' earnings. The staff consists of the Lady Superintendent, Miss Wells, who is highly praised by the committee, Nurse Vigors, for district nursing, and six private nurses. His Excellency, the Governor of the Island, spoke in favour of the institution.

HONOURS FOR THE FIRST THOUSAND.—An artistic certificate, designed by Miss Lillian C. Smythe, has been signed by the Princess of Wales, and will be presented to the First Thousand Nurses who joined the National Pension Fund, and thus secured to their comrades and to themselves the munificent donation of £20,000. The etching is extremely handsome, and is ornamented with an allegorical design showing how Rank and Benevolence reward Labour and Thrift. It is sure to prove a cherished possession of those nurses who are fortunate enough to possess it.

POPULAR NURSING.—A proof of the present popularity of nursing is found in a lecture lately delivered in the rooms of the Young Men's Christian Association at the Isle of Wight. The lecturer, Mr. Evelyn Rich, gave the usual general directions, which are not needed by a woman of common sense, and which a woman without common sense could never benefit by. "The art of nursing was to help the patient to live. The first recommendations for a nurse should be implicit obedience and discretion. A careful and subdued manner, and a pleasant voice under trying circumstances were essential, combined with firmness. Chattering was objectionable, although some patients were better for a little conversation. That had to be left to the discretion of a nurse. Let them never make light of anything a patient complained of, but endeavour to give a correct account to the medical man. The dress of the nurse should consist of washing material, and no noise should be made in walking. A pair of goloshes would prevent that." For our part we should think goloshes would be very bad for a nurse's feet, and that she might devise some more comfortable and beautiful foot-gear that would also be noiseless. We pity the patient who has to watch a nurse's feet clad in goloshes.

THOUSANDS OF POUNDS FOR THE NATIONAL PENSION FUND.—We have the pleasure to acknowledge the following sums towards the £14,000 now being raised to at once bring the Donation Bonus Fund up to £40,000. We hope to be able to acknowledge contributions amounting to £1,000 every week until the £40,000 are completed. Contributions of any amount may be sent to the Editor of The Hospital, or to the Manager, National Pension Fund for Nurses, 8, King Street, Cheapside, London, E.C.:

The Eighth Thousand.

| | | | | | | | |
|---|---|---|---|---|---|---|---|
| Messrs. N. M. Rothschild & Sons ... ... ... | £400 | 0 | 0 | Mr. P. A. Nairne ... | £25 | 0 | 0 |
| Messrs. Speyer Bros. ... | 105 | 0 | 0 | Mr. Charles Branch ... | 25 | 0 | 0 |
| Mr. H. Hoskier ... ... | 100 | 0 | 0 | Mr. Thomas Bryant ... | 25 | 0 | 0 |
| Mr. F. C. Hills ... ... | 100 | 0 | 0 | Mr. A. Lafone, M.P. ... | 25 | 0 | 0 |
| Messrs. Morton, Rose, & Co. ... ... ... | 100 | 0 | 0 | Mr. W. K. Foster ... | 10 | 0 | 0 |
| | | | | Mr. H. C. Saunders, Q.C. | 10 | 0 | 0 |
| Mrs. A. E. Rawlings ... | 30 | 0 | 0 | Mr. G. F. White... ... | 10 | 0 | 0 |
| Mr. H. M. Macpherson | 25 | 0 | 0 | Mr. E. Schwabacher ... | 5 | 5 | 0 |
| | | | | Mr. E. S. Boulter ... | 5 | 0 | 0 |

Amount already received **Eight Thousand Pounds.**

ELY DISTRICT NURSE.—There is only one district nurse at Ely, but she has a grand committee, and annual meetings all to herself. The Bishop of Ely presided at the last meeting, and said very complimentary things about the nurse, who has had a hard time of it during the late epidemic. The Ely District Nursing Association is in connection with St. John's House, Worcester.

YORK HOME FOR NURSES.—This Home has paid off its debt and started the present year with a balance in hand of £37. The staff during the past year consisted of the sister-in-charge, two sisters working amongst the poor, and 38 nurses and two district nurses. The increase of work has been so rapid that the council have determined to appoint five additional nurses, so that the number now employed is 45. During the past year cases had frequently to be refused, as the staff of nurses was quite inadequate to cope with the demands made upon it. The figures for 1889 were: Remunerative cases 457, gratuitous 465, total 922. The total for 1888 was 627. The Home is managed by the Sisterhood of the Holy Cross.

A MATRON'S GRUMBLE.—To grumble about grumbling is rather an illogical proceeding, but one to which an ex-matron has unkindly treated us. She says, "I never met with any of the foolish, carping women who now write irritable letters." Then she goes on to point out in what reads to us very like an irritable letter—that the modern trained nurse who goes to dances is an overpraised and flattered being who forgets her proper position. Be it noticed that our ex-matron acknowledges she is untrained. We do not admire grumbling, but it is only just that nurses should be allowed to express their opinions in a paper issued in their interests, and the ex-matron (whose letter is too long and too strong for publication) has surely exaggerated the number of our carping contributors.

THE SCARBOROUGH NURSES' INSTITUTE.—This institute was opened some two years ago, and has lately been transferred to much more commodious premises. The pleasant little house at first taken was found to be quite inadequate for the number of patients and for the nursing staff when at home. And although the owner of the house very kindly suggested enlarging the place, which could easily have been done, it was decided that it would be best to move a little nearer the town. Accordingly a very large house in Falsgrave Road has been chosen. It is very pleasantly situated, with a south aspect, and is in every way suitable for the purpose of receiving patients. During the past year considerably over 100 cases have been nursed, and it is very satisfactory to the superintendent to be able to say that not one bad or indifferent report has been received from the people employing the nurses, either doctors or patients. The Superintendent (Miss Edith Williamson), sister to Sister Dora of Scarborough, has always been most particular only to engage nurses who were thoroughly trained, most of them having been trained on the three years' system; all, with one or two exceptions, are certificated. Miss Edith Williamson has, of course, had the valuable help of her sister's advice and experience, who has been in the work without intermission for the last fourteen years. Many old friends joined them last summer for a holiday, so Miss Williamson conceived the idea that she would lay herself out to make the "home" a "house of rest" for nurses at the lowest possible charge. Picnics will be arranged, and pleasant little musical evenings, and no one need fear not having a good time at the "Scarborough Home Hospital," should they go there this summer for their holidays.

### The National Pension Fund for Nurses.

A BONUS FUND OF £40,000 IN SIGHT!

The third annual general meeting was held on Thursday, March 6th, at the offices, 8, King Street, Cheapside, Mr. Walter H. Burns in the chair.

The Report.

The report submitted set forth the following particulars: *Pension branch.*—During the six months there have been received 221 proposals (including 33 brought forward from last account) for granting pensions for £4,200 5s. 7d., of which 9 proposals, for assuring the sum of £245 were not proceeded with, or are still under consideration; 212 policies were granted, for assuring £14 13s. of immediate annuities, and £3,888 18s. 2d. of deferred annuities, producing £1,929 6s. 6d. in single payments, and £2,176 18s. in annual premiums; 1,039 policies have been issued from the commencement. *Sickness branch.*—In this department there have been received 45 proposals (including 8 brought forward from last account) for weekly sick-pay assurance of £32 10s.; six of these were declined or not carried out. 39 policies were granted, securing weekly sick-pay of £29 5s. 0d., producing annual premiums amounting to £53 7s. 0d. 198 Policies have been issued from the commencement. *Investments.*—The investments on the 31st December, 1889, amounted to £51,212 7s. 10d. as follows: In British Government Securities, £20,121 17s. 6d.; in Foreign Railway and other Debentures and Debenture Stocks, £22,067 15s. 6d.; ditto shares (Preference and Ordinary), £3,022 10s. 10d.; in Foreign Government Securities, £3,125 12s. 0d.; in Municipal Corporation Bonds and Stocks, £2,874 12s. 0d.—£51,212 7s. 10d.

The report also stated that the *Donation Bonus Fund* now amounts to £36,737 1s. 1d., and called attention to the fact that the managers of London and provincial hospitals were more and more realising the advantages of the Fund, and by way of encouraging nurses to be provident, were adopting the principle, on certain conditions, of paying half the premium of every nurse in their employment who joined the Fund. The council also stated that her Royal Highness the Princess of Wales had agreed to sign the certificates to be presented to the first thousand nurses in June or July next.

"An Uninterrupted and Steady Progress."

The Chairman (Mr. Burns) in proposing the adoption of the report and statement of accounts, said: In presenting the report of the Council to the third general meeting of the members of the National Pension Fund for Nurses, there seems very little to be added by me in explanation of the accounts. They are so full and explicit that they speak for themselves. The progress of the fund has been uninterrupted and steady since July 1st last, and you will see that in all departments there has been a regular increase of business, and that the contributions of the public to the Donation Bonus Fund have been very considerable. As the accounts show upwards of £32,000 had been received for that purpose up to December 31st, and I may add that additional subscriptions made since that date have raised it to upwards of £38,000, making at the present moment, with the Annuity and Sinking Fund and other contributions over £60,000 contributed to the purpose of the Association. Of this amount £5,000 were given by members of the Stock Exchange. We have every reason to believe that the sum of £40,000, which the members of the Council hoped to obtain for the Donation Bonus Fund, will be reached, if not exceeded, before many weeks have elapsed. I think it will be well for me to mention with regard to the accounts rendered to June 30th, 1889, that they seem to have left in the minds of some parties a slight uncertainty as to the actual expenses incurred in founding the company. The costs of management were set down as £762 10s. 3d., and in addition there was a sum of £1,287 10s. expended, which was met by special donations. This latter sum represents what was spent during the many months, and we may say years, of preliminary work, lengthy correspondence, and numerous circulars addressed to various parties, and all the work of gathering statistics, actuaries' fees, rent, salaries, and disbursements of all sorts up to the period when the company actually received its charter and issued its first policies, which latter was not until July, 1888. The sum of £762 10s. 2d. represented the expenses of managing the company from the time we commenced issuing policies, about July 1st, 1888, being the period when the Association was actually in corporate existence, and when the expenses became properly chargeable to the Nurses' Fund. The donors of the £1,287 10s. made this gift specially in order that none of the preliminary charges should fall upon the policy holders. I trust that this will render the subject entirely clear to all interested, and that the nurses will understand and appreciate the fact that not one penny of the preliminary expenses has come out of their payments or pockets, in any shape whatever.

The Fund's First Legacy.

I am pleased to be able to announce that we have received a communication from the notaries, Messrs. Ormrod and Allen, of Manchester, that under a provision in the will of Mr. Daniel Proctor, deceased, a grant of £50 has been made to the National Pension Fund. I mention this specially, not on account of the amount, which is not important, but because it is the first legacy which we have received, and I trust that the example thus set will lead many other charitable and well disposed people to insert the name of our institution among those legatees to whom they may be making bequests.

Active Personal Interest of the Princess.

In moving the adoption of the report, I cannot conclude without expressing our thanks to H.R.H. the Princess of Wales, for the active personal interest she has taken, and continues to take in our Association, and I am sure it must be a cause of great pride and satisfaction to the nurses and annuitants, as well as to the members of the Fund.

Mr. Henry C. Burdett (deputy chairman and founder of the Fund): I second the resolution, and in doing so would point out that the progress of business is satisfactory. At the present time, without any special efforts, we are issuing policies at the rate of one per diem. It is also satisfactory to note that, although the Donation Bonus Fund now approaches £40,000, the whole of that sum has been raised without the expenditure by the Council of any money whatever for postage, stationery, or matters of that kind. Practically, it has all been given spontaneously to the Fund through members of the Council.

The resolution was adopted.

Council and Officers.

The Chairman explained that the five members of the Council retiring by rotation were eligible for re-election. Mr. F. C. Carr-Gomm, however, had written to him to say that he had not time to devote to the duties, and so did not desire to be again appointed. Mr. E. Murray-Ind, chairman of the London Hospital, would be prepared to accept a seat on the Council if elected by this meeting. He accordingly proposed that Mr. E. Murray-Ind be appointed, and that the following gentlemen be re-elected: Alfred de Rothschild, Esq., Dr. J. C. Steele, Edward Rawlings, Esq., and Clifford Wigram, Esq.

Mr. Hugh McPherson seconded.

Passed unanimously.

On the proposal of the Chairman, seconded by Mr. Burdett, the auditor, Mr. Fredk. Whinney (Messrs. Whinney, Hurlbatt, and Smith, chartered accountants) was reappointed.

A vote of thanks to the chairman concluded the proceedings.

### Between Cases.

Your duty to your patient is over, and you leave a face bright with renewed health or white and calm with the last sleep, and go back to the little room in which—if you work on your own account—you have made your home. Perhaps you had taken the key with you, or had not time to advertise the landlady that you were coming back, so there is no fire in the little grate, and the dust of weeks rises to welcome you. But still you are here in your own place, and no one will call or bother you. You can sleep, walk, eat as you will. Oh liberty! Blessed liberty! Only a woman who for weeks has been in constant attendance in a sick room; who for many nights has slept the light sleep that listens for a whisper or a movement; who has had to walk warily lest over-zealous friends or old servants take offence, knows the rest and peace of those first few hours. Then you dine off a new loaf and tinned lobster, or anything else easily procured, and make tea in your very own teapot, and sit with your feet in the fender reading the last number of The Hospital, happy for a time because you are free.

After a day or so, you call on the doctors and then you sit at home and wait, and time hangs heavier each day. You begin to feel depressed because there is no one to talk to, and you are not properly fed, so that when a case turns up you are often less fit for work than you should be. Nor are the nurses attached to a nursing institution better off. Certainly we know of one little home where they are made happy during the intervals. But as a rule the institution nurse has fewer comforts than the woman who is on her own account. The former shares a room with several others, and she cannot well adorn her portion of it with the little ornaments and photos that make the barest wall pleasant to look at, because she is liable to be summoned away in haste, and her treasures may not be left to the mercy of the next comer.

Considering the difference between the earnings and the salaries of the nurses, it is curious that the institutions can. not always manage to give their staffs a comfortable sitting-room, but, as yet, easy chairs and sofas are not common, and do any of the private homes provide the nurses with a piano? It may be said that as many of the institutions combine patients and private nurses, the former would be disturbed by a piano, but this is only a reason why such combinations should not be encouraged. The laughter that cheers is very subdued in these places, and again, if the indoor staff is short-handed, the nurse who returns from a hard case and needs a rest does not always get it.

Those who get the best time between cases are the little bands of wise nurses who combine six or eight together to support their own small establishment. They rent a flat or a small house, and usually one of the members has a sister or friend who undertakes to look after the home-comforts and interests. These friends are happily certain that letters will be punctually forwarded, and calls and inquiries reported; they are also free from the fear that their favourite doctor will waste ten minutes of his valuable time on the doorstep before the careless landlady will trouble to answer their bell. And what expeditions they can make when two or more are disengaged at the same time! Some of us will always remember the impromptu jaunts to Windsor, to Epping and Hadley Woods, in company with sausage-pies and cakes of our own special make. As for the curious folks we have met when on these excursions, and the remarkable adventures that have occurred, are they not chronicled in our memories, and still spoken of lovingly, when again together we sit over the fire and exchange confidences in that sympathetic hour before going to rest?

We hear much of the co-operative nurses. Why should they not combine for pleasure as well as profit? It will be so easy by means of their register to find out whether a comrade is at liberty to join in a small tea party, or a picnic. Then the members of "Our Own" can take their holidays with a quiet mind, peacefully conscious that their interests will be well looked after at their office, and that they will lose nothing by a fortnight spent at the seaside, or a little trip to the Continent.

### Everybody's Opinion.

*[Correspondence on all subjects is invited, but we cannot in any way be responsible for the opinions expressed by our correspondents. No communications can be entertained if the name and address of the correspondent is not given, or unless one side of the paper only be written on.]*

### THE TITLE OF NURSE.

A Nurse writes: At a large public meeting in this town, a lady delegate, speaking of infant insurance by parents, said it as though nurses were given a direct interest in the death of their charges, and she added an anecdote of a nurse who was severely censured for effecting an insurance on the life of a patient in the institution to which they both belonged. As this savoured somewhat of "St. Bernard's," and as the idea of nurses speculating in the lives of their patients certainly adds a new terror to sickness, I ventured, during the interval for tea, to question the speaker, and was informed that she had read the story somewhere, but had been unable to verify it, that she was pretty sure it did not occur in any hospital or sick infirmary, and finally that the offender was not a nurse, but a male attendant. Our sisterhood being thus far vindicated, I merely wish now to protest against the use of the term "nurse" for everyone who has to do with, or do for, the sick. More especially it should not apply to men. Let them be sick attendants, hospital orderlies, if they please; nurses they are not, and cannot be. Nature, etymology, and common sense alike forbid it.

### MEUM AND TUUM.

Handclasp writes: Justine's letter can scarcely fail to excite the attention of all who may chance to read it. It not only affects the management of wards, the treatment of patients, but the character of the nurses in charge of them. We have had endless complaints from nurses all last year about one thing or another, low salaries, overwork, bad food, harsh treatment, long hours, &c., but now we have a distinct complaint against the nurse herself, and that by one of ourselves. Does it remind one of the "cart before the horse?" Perhaps it is not very clear to the reader whether Justine complains of the treatment she and her friend received as nurses, or of the nurses who could so far forget their office as to treat any suffering being in the manner stated. It is time we took this bull by the horns when one of our number tells us we "can calmly and unblushingly tell an untruth to a helpless sufferer in order to quiet him or her," and apparently Justine thinks it "particularly hard when the patient happens to be a nurse." To me this seems the very acme of selfishness, and one can imagine the feelings of any patient who may read it. Will he not cry out with Shylock, "Am not I suffering? Have not I hands, eyes, organs, dimensions, senses, affections, passions like unto you, O! nurse? fed with the same food, hurt with the same weapons, warmed and cooled by summer and winter? If you prick us do we not bleed?" But as long as nurses enter hospitals merely to gain a respectable living, and not for love and devotion to the sick, such pitiable instances of gross neglect and wicked indifference will not be uncommon. What we need is a combination of nurses faithful and true, who should not only by their own example live down, but root out such unworthy specimens of womanhood. Ought we to tolerate evil for fear of the consequences? Would it have been fair to the "good kind surgeon" if his efforts for Justine's relief had failed, and her weak connivance with wrong on the part of her nurse or dresser had resulted in serious inflammation, perhaps ending in gangrene and death for fear of hurting his feelings or the nurse's reputation? I grant it is harder to speak up for the right, when the wrong only affects ourselves, and one's motives may be misconstrued, and we are told we are ungrateful, etc.; but should anything deter us from the upward, onward course? What is the consequence in the cases before us? Nurses who are not only unfaithful, neglectful, but who lie to conceal their faults are probably still palmed off on unsuspecting victims as good nurses, damaging like rotten fruit a bowlful of the sound. Charges of so grave a nature when supplied anonymously effect no good in exterminating the evil, only serve to lessen respect and deepen distrust in the public mind. I am far from wishful to defend probationers, "who, robed in a little brief authority" misuse it, yet I have found that when they are rude, curt, and unnurselike, the evil may be traced to those over them. "Like clings to like," and the sister who is gentle and firm will generally be found to have quiet orderly probationers under her rule. The question raised by Justine as to the advisability of calling patients by the number of their beds or by their own names is one open to discussion. Whilst inclining theoretically to the latter, practically the former method greatly facilitates the administration of large double wards, and possibly fewer mistakes may arise in giving orders to probationers. A little kindly explanation to each new patient is all that is needed. The difficulties of taking "charge" of a ward for the first week are not a little enhanced by the effort to retain in one's memory, not only the various diseases and their treatment, but the names of some thirty or more patients, and in female wards confusion is worse confounded by the married and single being equally tenacious of their rights. I cannot help thinking that in those hospitals where numbers are not in use, a practice too frequently arises of speaking of the patients by whatever complaints they may be suffering from; and whether the sufferer prefers to be designated by his disease or a number, I will leave others to decide. Why is it more objectionable for a patient to be called after a number than for a nurse or "sister" to sink her own name in that of her ward? Justine must know from experience how impracticable it is to treat one patient differently from another if nursed in the general wards. It creates ill-will, discontent, and jealousy, incompatible with the order and well-being of a ward, but no one should be allowed to feel himself or herself a "nonentity," hurt and wounded by the very instrument provided by the public to administer to his or her comfort. Everyone besides a nurse "will find a kind word help them in their severe trials." If the fact of a human being's sufferings, whether he be a poor cowering idiot or the most accomplished man living, does not appeal to the highest instincts of a womanly nurse, then I doubt whether anything will appeal, not even though she be a nurse. What can I say more than that our whole duty is comprised in the golden rule—"Do unto others even as ye would they should do unto you."

### Appointments.

[It is requested that successful candidates will send a copy of their applications and testimonials, with date of election, to The Editor, The Lodge, Porchester Square, W.]

Leeds General Infirmary. — Miss Fisher has been appointed superintendent of nurses at this infirmary. Miss Fisher has been for some time assistant superintendent at Leeds, so is familiar with the work; previously she was night sister at Liverpool Royal Infirmary.

Asylums Board's Eastern Hospital.—Miss Ida Dewing has been appointed as matron of the above hospital. She previously held the appointment of matron at the Stafford County Infirmary.

Dartmouth Cottage Hospital.—Miss F. E. Cole, who trained at Guy's, has been appointed matron of this hospital. Miss Cole has lately been working at Rickmansworth, and her departure causes much sorrow to many of those she has nursed there.

St. Albans Hospital.—Miss Evelina Bishop was lately appointed matron of this hospital. She trained at the Royal Albert Edward Infirmary, Wigan, and became charge-nurse of the Gidlow Wards there, and finally assistant-matron. There were 100 applicants for the matronship of St. Albans.

Western General Dispensary.—Miss Kate Heathcote has been appointed matron of this dispensary in the Marylebone Road. Miss Heathcote trained for two years at Pendlebury, was for two years staff nurse at St. Bartholomew's, and for three years head of the Albert and Leopold wards at the Royal United Hospital, Bath. In addition to medical and surgical nursing, Miss Heathcote is experienced in fever, obstetric, and ophthalmic work. Her testimonials are good.

Erratum.—It should have been stated last week that Miss E. Nugent acted as *locum tenens* head nurse at the Paulton Hospital for three months, and not that she actually filled that post permanently.

### Amusements and Relaxation.

### FOURTH QUARTERLY WORD COMPETITION

Commenced Jan. 4th, 1890, ends March 29th, 1890.

Three prizes of 15s., 10s., 5s., will be given for the largest number of words derived from the words set for dissection.

Proper names, abbreviations, foreign words, words of less than four letters, and repetitions are barred; plurals, and past and present participles of verbs, are allowed. Nuttall's *Standard* dictionary only to be used.

N.B.—Word dissections must be sent in WEEKLY, arranged alphabetically, with correct total affixed.

The word for dissection for this, the ELEVENTH week of the quarter, being

"DAFFODILS."

| Names. | Mar. 6. | Totals. | Names. | Mar. 6. | Totals. |
|---|---|---|---|---|---|
| Holland | 159 | 864 | Nurse Clague | — | 40 |
| Red Cape | — | 132 | Lauriston | 159 | 873 |
| Nurse Marie | — | 499 | B. E. T. | — | 453 |
| Garfield | — | 243 | Jacko | — | 110 |
| Midget | 96 | 754 | Lady Clara | — | 258 |
| E. M. S. | — | 149 | Patience | 168 | 910 |
| Sister | — | 591 | Ecila | 169 | 899 |
| Duchess | 89 | 585 | Maude | — | 690 |
| Ellice | 153 | 868 | Esperance | 169 | 892 |
| Reldas | 171 | 908 | Ruby | — | 698 |
| Jenny Wren | 132 | 785 | M. W. | 168 | 891 |
| Mopus | — | 122 | Crystal Palace | — | 568 |
| Neptune | — | 118 | Jackanapes | 102 | 661 |
| Qu'appelle | 135 | 825 | M. M. M. | — | 25 |
| Whitehoe | 138 | 791 | A. F. | — | 318 |

### Notice to Correspondents.

N.B.—All letters referring to this page which do not arrive at **140, Strand, London, W.C.,** by the *first post on Thursdays*, and are not addressed PRIZE EDITOR, will in future be disqualified and disregarded.

### Vacancies.

The following vacancies are announced:

### Matrons.

Colchester Hospital; £60.
Clayton Hospital, Wakefield; £50.
Gorleston Cottage Hospital; £35.

### Nurses.

Abbey Poorhouse, Paisley; £22.
Aimee Nursing Home.
Bournemouth Victoria Home; £25.
Burton-on-Trent Infirmary; £25.
Bury St. Edmund's Hospital; £25.
Bournemouth Institution; £25.
Bedfordshire Institution, £23.
Bury Dispensary Hospital; £25.
Benefit Nursing Association, Reigate.
Coventry Hospital (night); £20.
Cardiff Infirmary; £24.
Chester Deaconess House; £25.
Croydon Union; £22.
Cambridge Home for Nurses.
Devonport Albert Hospital; £25.
Devon and Exeter Hospital, £24.
Ear and Eye Infirmary, Liverpool; £20.
Eastbourne Sanatorium; £30.
Edinburgh Poorhouse; £25.
Fitzroy House (Masseuse).
Glasgow Bornhill Hospital; £24.
General Nursing Institute.
Glasgow Hillhead Home; £30.
Hull Nurses' Home.
Harding Nursing Institute.
Hampstead Home Hospital.
Johnson Hospital, Spalding; £20.
Kendal Hospital; £28.
Leeds General Infirmary (superintendent); £50.
Liverpool City Hospital; £25.
Mr. Wilson's Institution.
Middlesbrough Association.
Mile End Workhouse (night); £25.
North-West London Hospital; £22.
Newlands, Ryde; £25.
Newport Nursing Institute.
Nottingham and Notts Nursing Association; £25.
Northampton Nursing Institution; £25.
Pendlebury Hospital; £20.
Poole Cornelia Hospital.
Paris; Miss Neilson; £28.
Queen Victoria Nursing Institute.
Royal Chest Hospital; £22.
Royal Portsmouth Hospital (Lock); £25.
St. Saviour's Infirmary; £20.
Shoreditch Infirmary; £20.
St. Leonard's Nurses' Home.
Southport Nurses' Home.
Stamford, Rutland, and General Infirmary; £25.
Stroud Hospital; £15.
Southport St. John's Institute; £27.
Swansea Institute; £25.
Tunbridge Wells Institute; £30.
Victoria Park Hospital; £20.
West Bromwich Hospital; £22.
West London Hospital; £28.
Workhouse Nursing Infirmary Association.

### District Nurses.

Burton-on-Trent; £25.
Great Bedwyn, Wilts; £25.
Leeds District Nurses' Home; £25.
Wey Springs, Haslemere.

### Probationers.

Bristol Royal Infirmary.
Birmingham Skin Hospital.
Burton-on-Trent Institution.
Dewsbury Infirmary; £10.
Guildford County Hospital.
Kent Nursing Institution.
Newport Infirmary.
Nottingham and Notts Nursing Association.
Nottingham Samaritan Hospital.
Southport Massage Institution.
Torbay Hospital.
Workhouse Nursing Infirmary Association.

### Notes and Queries.

### Queries.

(62) *Home Wanted.*—For a lady, age 63, who has had a stroke, and whose heart is weak. She is utterly destitute, but friends would pay a small sum, or would it be possible to secure an allowance for her from any charity?—*C. M. F.*

(63) *Obstetrical Nursing.*—Where can I learn both general and obstetrical nursing at the same time?—*E. M. W.*

(64) *Uniform Cloaks.*—Is there any special shop where nurses' cloaks can be bought?—*Nurse E.*

(65) *Uniform Caps.*—Is there any shop where I can send for ready-made patterns of caps to choose from?—*C.*

(66) *Cleanliness.*—Which is the best way to effectually clean live heads without injury to the hair, and is there any prevention against the occurrence of this evil?—*Nurse.*

(67) *Macintoshes.*—How can I remove stains from macintoshes?—*A.*

(68) *Coffee Stall.*—I shall be much obliged to anyone who can tell me the cost of a coffee stall for out-patients at a hospital, and the management generally. I see the Great Northern Central Hospital has just started one.—T. M. B.

### Answers.

(55) *Nourishing Drinks.*—1. Raw meat pulped through a sieve and given in beef tea. Must on no account be cooked, or will become gritty, but carefully rubbed down by degrees and given lukewarm. 2. Whites of eggs well beaten and mixed with beef tea. 3. The same, also well beaten and mixed with milk, straining after mixing. All these can be given without annoyance if properly done. Also raw beef tea prepared at home, or Valentine's meat juice. If needed, will give fuller directions for preparation.

(58) *Rolling Bandages.*—I advise Mrs. B. to get the Universal Bandage Winder from Reynolds and Branson, 13, Briggate, Leeds. Price, post free, 2s. 9d.—*Nurse Mina.*

(63) *Obstetric Nursing.*—Under the Workhouse Infirmary Nursing Association, 6, Adam Street, Strand, for one place, and there are many others.

*Kirkham and M. G.*—Male nurses are trained by the Hamilton Association, 57, Park Street, Grosvenor Square, W. Massage can be learned at 73, Welbeck Street, W.

*Massage.*—See answer to *Kirkham.* Also apply to Mrs. Creighton Hale, 46, Madox Street, W.

*Examinations.*—We beg to inform Nurse G. that there is no central place where she can be examined; her testimonials must stand her in stead of a certificate. Nurses should try and not enter at a hospital where no theoretical training and no certificates are given.

*Army Nursing.*—"Vivandiere" must train two years in a good general hospital, and then apply to the Director-General of the Army Medical Department, Victoria Street, Westminster.

*H. S.*—Your query could only appear as an advertisement.

"*Musical Nurse*" must send name and address.

